# The role of lipoprotein profile in depression and cognitive performance: a network analysis

**DOI:** 10.1038/s41598-020-77782-9

**Published:** 2020-11-26

**Authors:** Qiu-fang Jia, Han-xue Yang, Nan-nan Zhuang, Xu-yuan Yin, Zhen-hua Zhu, Ying Yuan, Xiao-li Yin, Yi Wang, Eric F. C. Cheung, Raymond C. K. Chan, Li Hui

**Affiliations:** 1 The Affiliated Guangji Hospital of Soochow University, Medical College of Soochow University, Suzhou, Jiangsu People’s Republic of China; 2 Neuropsychology and Applied Cognitive Neuroscience Laboratory, CAS Key Laboratory of Mental Health Institute of Psychology, Beijing, People’s Republic of China; 3 Wenzhou Kangning Hospital, Wenzhou Medical University, Wenzhou, Zhejiang People’s Republic of China; 4 Castle Peak Hospital, Hong Kong, People’s Republic of China; 5 Department of Psychology, University of Chinese Academy of Sciences, Beijing, People’s Republic of China

**Keywords:** Psychiatric disorders, Lipids

## Abstract

Lipid profile (total cholesterol and lipoprotein fractions) has been found to correlate with depression and cognitive impairment across the lifespan. However, the role of lipid levels in self-rated depressive state and cognitive impairment remains unclear. In this study, we examined the relationship between lipid profile (total cholesterol, triglycerides, high-density lipoprotein cholesterol and low-density lipoprotein cholesterol) and cognition in adults with and without self-rated depression. Four hundred and thirty-eight healthy participants completed the Repeatable Battery for the Assessment of Neuropsychological Status (RBANS), the Self-Rating Depression Scale (SDS), and a serum lipoprotein test. Using multivariate ANOVA, partial correlation and network analysis, a network linking lipoprotein profile, depressive state and cognition was constructed. A significant difference in serum lipid profile between the high and low depressive groups was detected. Depressive state had a strong negative correlation with cognitive performance. Of the lipid profile, only high-density lipoprotein was positively correlated with depressive symptom severity, whereas the other three indices showed negative correlation with both depressive state and cognitive performance. Our results suggest that serum lipid profile may be directly linked to self-rated depression and cognitive performance. Further studies recruiting larger clinical samples are needed to elucidate the specific effect of lipoprotein on cognitive impairment in mood disorder.

## Introduction

Depression is characterized by significant cognitive impairment^1,2^. Previous research has shown that serum lipid levels are associated with cognitive function, although some of the results are mixed. Evidence suggests that high-density lipoprotein (HDL) may be able to boost cognitive function^3–6^, with a more pronounced effect on memory among women^7^. As for total cholesterol (CHO), low-density lipoprotein (LDL) and triglycerides (TG), some have observed that higher CHO^8^, higher LDL^7,9^ and lower TG^8,10^ are associated with better function in most cognitive domains, whereas other studies have found no significant correlation between these three lipoproteins and cognitive function^11,12^, or an opposite effect^8,13^. Also, a detrimental role of CHO on memory and hippocampal morphology has been reported in an animal study^14^. Notably, a direct pathway from serum lipid levels to cognitive impairment in depression has also been reported^15^, but the above four serum lipid indices have been suggested to have somewhat different roles. For instance, a Chinese population-based study concluded that only higher TG level contributed to greater cognitive impairment in major depressive disorder, especially in delayed memory^15^.

The role of serum lipid levels in depression has also been established in elderly men^16^, middle-aged men^17^, young adult women^18^, and general population across the lifespan^19^. Yet conclusions regarding the correlation between serum lipid profile and depressive symptoms are inconsistent, ranging from direct to inverse and sometimes no correlation^20^. An inverse relationship between depressive symptoms and higher CHO level^21–23^ and higher LDL level^18,24^ have been reported. One study reported that low HDL and high LDL levels may be correlated with more severe depressive symptoms in a Chinese sample^25^, while another study has found that depression may be correlated with low LDL level rather than HDL level^26^. Drawing on their own results, Igna and colleagues proposed an “unfavourable serum lipid profile” characterized by higher HDL level, lower LDL and CHO levels^24^. In addition, they suggested that age, education level, smoking habit and body mass index (BMI) are key mediating factors that have significant negative impact on decreased HDL and increased LDL levels^18,24^. Pan et al.^27^ also found significant correlations between individual components of the metabolic syndrome (CHO and TG) and depression in their meta-analysis on the bidirectional association between depression and metabolic syndrome. More recently, a meta-analytic review has linked depression with overall lower serum LDL level, though high heterogeneity was also noted^28^.

The aim of this study was to examine the relationship between lipoprotein profile (CHO, TG, HDL, and LDL) and cognitive function in adults with and without self-rated depression. Most previous studies that explored the relationship between serum lipid levels and depression used multiple linear regression analysis after adjusting for confounding factors^29^. Here we adopted the network analysis approach, a new visualization tool that takes into account partially controlled correlational links among conceptualized variables^30^. We hypothesized that: (1) in general, significant negative correlations would be present between depressive state and cognitive performance, while serum lipoprotein levels would associate positively with depressive state and negatively with cognitive performance; (2) people with high and low self-rated depressive state would differ in cognitive function and serum lipoprotein levels; and (3) major demographic and lifestyle confounding factors (i.e. age, sex, education level, smoking status and BMI) would have a significant effect on serum lipoprotein levels, cognitive function, depression status and their relationships.

## Results

### Sample characteristics and group differences

Table 1 summarizes the demographic, lipoprotein and cognitive information of the sample. The Self-Rating Depression Scale (SDS) total score did not differ between male and female participants after controlling for age. There was no significant difference in sex, education level, smoking status, BMI, and lipoprotein levels between the low-depressive (LD) and high-depressive (HD) groups. Although we sought to recruit individuals with and without depressive symptom in the study, all participants with a history of any physical and mental illness including major depressive disorder, alcohol and drug dependence were also excluded. Therefore, only a small part of the participants was classified into the HD group. The HD group was significantly older than the LD group (F = 2.17, *p* = 0.03). However, all lipoprotein levels except HDL were found to be slightly higher in the LD group compared with the HD group. This pattern was reaffirmed in the subsequent multivariate ANOVA after controlling for confounding factors, in which significant discrepancies in CHO (F = 3.821, p = 0.002), TG (F = 15.649, *p* < 0.001), LDL (F = 3.654, 0.003) and HDL (F = 7.606, *p* < 0.001) levels were detected between the two groups. As for cognitive function, the LD group showed significantly higher scores on the Repeatable Battery for the Assessment of Neuropsychological Status (RBANS) except for the “language” subscale compared with the HD group. Moreover, after controlling for confounding factors, all sub-scale scores differed significantly between the two groups (see Table 2). Additionally, in our whole sample, BMI did not show any significant correlations with either depressive state (r = 0.018) or total RBANS score (r = −0.079).

**Table 1:** Characteristics of participants.

Characteristics	Total sample (N = 438)
Age (years), mean (SD)	34.83 (1.09)
Gender, female% (n)	71.2 (312)
Education (years), mean (SD)	12.74 (3.29)
BMI, mean (SD)	22.09 (2.89)
**Smoking, % (n)**	
No	84.5 (370)
Former	2.3 (10)
Current	13.2 (58)
CHO, mmol/L, median (IQR)	4.86 (1.22)
HDL, mmol/L, median (IQR)	1.47 (0.42)
LDL, mmol/L, median (IQR)	2.75 (1.05)
TG, mmol/L, median (IQR)	1.37 (0.85)
**RBANS total score, mean (SD)**	85.71 (13.13)
Immediate Memory	83.25 (16.24)
Visual-spatial/Constructional span	77.80 (13.41)
Language	92.41 (15.17)
Attention	105.26 (17.91)
Delayed memory	87.42 (11.28)
SDS standardized total score, mean (SD)	38.83 (8.66)

*BMI* body mass index, *CHO* total cholesterol, *HDL* high-density lipoprotein cholesterol, *LDL* low-density lipoprotein cholesterol, *TG* Triglycerides, *RBANS* Repeatable Battery for the Assessment of Neuropsychological Status, *SDS* Self-rating Depression Scale, *SD* standard deviation, *IQR* inter-quartile ranges.

**Table 2:** Cognitive performance and lipoprotein levels were significantly higher in the LD group than HD group after adjusting for the confounding factors.

Indexes	LD (n = 379)	HD (n = 59)	F (t)	*p*	Adjusted F	Adjusted *p*
Age (mean, SD)	35.27 (10.77)	31.98 (11.04)	2.17	0.03*	**–**	**–**
Gender (female%)	70.2	78.0	− 1.23	0.22	**–**	**–**
Education (years)	12.83 (3.19)	12.17 (3.85)	1.45	0.15	**–**	**–**
BMI (mean, SD)	22.04 (3.00)	22.44 (3.56)	− 0.94	0.35	**–**	**–**
**Cognitive function**						
Immediate memory	83.95 ± 16.04	78.79 ± 16.91	5.279	0.022*	10.53	< 0.001**
Visual–spatial/constructional	78.51 ± 13.47	73.22 ± 12.17	7.984	0.005**	7.47	< 0.001**
Language	92.79 ± 15.03	89.96 ± 15.96	1.756	0.186	8.06	< 0.001**
Attention	106.16 ± 17.40	99.47 ± 20.13	7.103	0.008**	22.44	< 0.001**
Delayed memory	88.18 ± 10.68	82.58 ± 13.65	12.950	0.000**	5.92	< 0.001**
RBANS total score	86.54 ± 12.72	80.44 ± 14.63	11.243	0.001**	19.15	< 0.001**
**Lipoprotein levels**						
CHO (mmol/L)	5.01 ± 1.01	4.85 ± 0.77	1.242	0.266	3.821	0.002**
TG (mmol/L)	1.62 ± 0.91	1.57 ± 0.88	0.173	0.678	15.649	< 0.001**
HDL (mmol/L)	1.48 ± 0.35	1.54 ± 0.32	1.192	0.276	7.606	< 0.001**
LDL (mmol/L)	2.81 ± 0.90	2.66 ± 0.61	1.680	0.196	3.654	0.003**

*LD* low depressive, *HD* high depressive.

Adjusted F, *p* values were controlled for age, gender, smoking and education years; *p < 0.05, **p < 0.01.

### Network estimation, centrality, network accuracy and network stability

**Figure 1:**
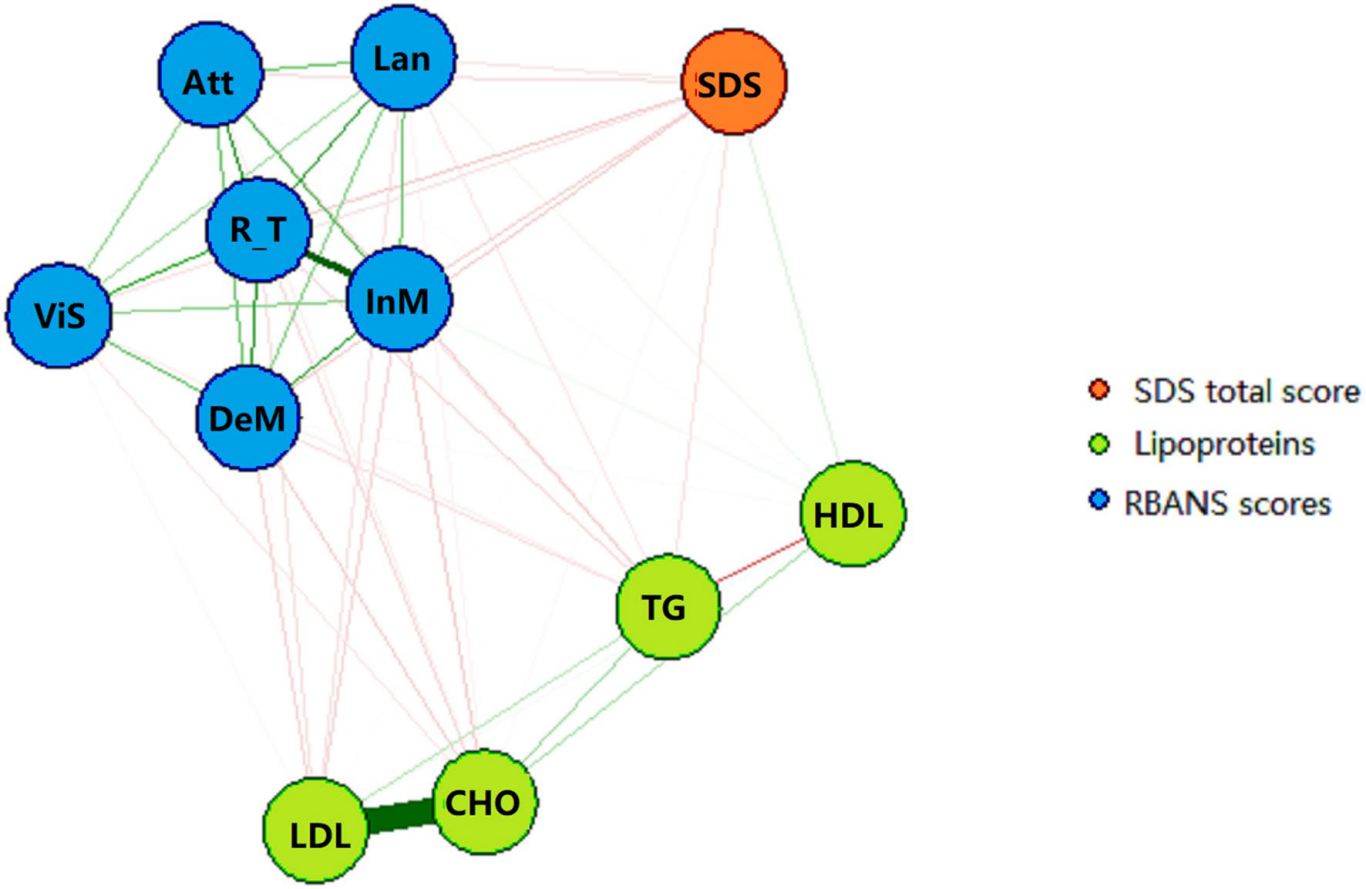
Network estimation of the whole study sample (N = 438). *Notes*: Red edges indicate negative correlations, green edges indicate positive correlations, with thicker ones representing stronger partial correlations. *Att* Attention sub-scale of the RBANS, *InM* Immediate Memory sub-scale of the RBANS, *ViS* Visual spatial/Constructional sub-scale of the RBANS, *Lan* Language sub-scale of the RBANS, *DeM* Delayed Memory sub-scale of the RBANS, *R_T* RBANS total score, *CHO* total cholesterol, *HDL* high-density lipoprotein cholesterol, *LDL* low-density lipoprotein cholesterol, *TG* triglycerides, *SDS* total score of the Self-Rating Depressive Scale.

A network of 11 nodes representing depressive state (SDS total score), lipoprotein levels (CHO, HDL, LDL and TG) and cognitive performance (immediate memory, visuospatial/constructional, language, attention, delayed memory, and RBANS total score) was constructed. As seen in Fig. 1, the community detection function identified three major communities, with depressive state (SDS total score) in red, lipoprotein indices (CHO, TG, LDL and HDL) in green, and cognitive performance (RBANS scores) in blue. Edges linking nodes within each community were the strongest, suggesting robust measurement validity. The nodes R_T (standing for “RBANS total score”) and TG (triglycerides level) had the largest number of edges, and were situated in the center of the cognitive and serum lipoprotein communities respectively. In general, the node SDS had a prominent negative link with the cognitive community, suggesting adverse effect of depression on adult cognitive performance; whereas edges from the serum lipid community had both positive and negative connections with the other two communities. Firstly, LDL, CHO and TG levels were all negatively connected with the cognitive performance community. Secondly, TG level had a similar negative link to the SDS node, while CHO and LDL levels were irrelevant to depressive state after controlling for all other nodes. Only HDL level was positively connected to both self-rated depressive state and cognitive performance. Intriguingly, TG and CHO levels were positively linked as one “unit” or “axis”. Within the lipid community, LDL and HDL each had one positive and one negative edge with this axis. LDL exhibited a strong positive connection to CHO and a weaker negative connection to TG. Inversely, HDL had a strong negative connection to TG and an equally strong but positive edge with CHO.

Network inference measures showed that the SDS node had the highest standardized strength centrality in the network, followed by R_T, both of which also had the highest closeness estimate in the network, suggesting that they had the shortest path connecting them to other nodes. CS coefficients were 0.594 for strength, 0.516 for closeness, and below 0.25 for betweenness. Since the main indicator of stability was the CS coefficient of strength, which was above 0.5, it was safe to assume that the current model was stable. The EI was at the maximum obtainable value of 0.75^31^. Additionally, results of the bootstrapped difference test, the edge weight accuracy test, and the edge weight difference test can be found in Supplementary Materials.

### Correlations between lipoprotein profile, cognitive performance and depressive symptom

Table 3 shows that for the LD group (n = 379), CHO, TG and LDL all had significant negative correlations with cognitive scores. Specifically, scores on the memory related sub-scales (i.e. “Immediate Memory” and “Delayed Memory”) were inversely correlated with CHO and LDL levels, while “Attention” was negatively correlated with TG level. Of the lipid profile indices, the correlation of HDL with cognitive performance was not significant. For the HD group (n = 59), TG had a moderate to strong correlation with score on the “Attention” sub-scale of the RBANS (r = −0.317, *p* < 0.05), while all other lipoprotein levels’ correlation indices were not statistically significant.

**Table 3:** Correlations between lipoprotein levels, TG, and cognitive performance across the LD and HD groups.

	LD (n = 379)				HD (n = 59)			
	CHO	TG	HDL	LDL	CHO	TG	HDL	LDL
Immediate memory	− 0.153**	− 0.076	0.026	− 0.140**	− 0.082	− 0.121	0.059	0.001
Visual spatial/constructional	− 0.059	− 0.048	0.018	− 0.017	− 0.168	− 0.021	− 0.049	− 0.018
Language	− 0.014	− 0.034	0.022	− 0.081	− 0.178	− 0.185	0.143	− 0.137
Attention	− 0.052	− 0.103*	0.007	− 0.032	− 0.026	− 0.317*	0.064	0.102
Delayed memory	− 0.159**	− 0.087	0.009	− 0.162**	0.017	− 0.073	− 0.045	0.091
RBANS total Score	− 0.113*	− 0.093	0.027	− 0.114*	− 0.089	− 0.190	0.062	0.024

Partial correlations, controlling for age, gender, smoking and education years, 2-tailed, *p < 0.05, **p < 0.01.

## Discussion

The present study examined the relationships among serum lipoprotein profile, cognitive impairment and self-rated depression using network analysis. The constructed network demonstrated the negative correlations of serum LDL, CHO and TG levels with cognitive performance. Moreover, TG level was negatively linked to depressive state, while HDL level was positively connected to both self-rated depressive state and cognitive performance. Group comparison revealed that the HD group exhibited lower levels of serum LDL, CHO, and TG, higher level of serum HDL, and performed significantly worse in cognitive tests compared with the LD group.

Prior research suggests that cognitive impairment may be a core feature of self-rated depression^15^. In general, we found that self-rated depression had a significant negative impact on cognitive performance. Lipoprotein profile also differed significantly between the HD and LD groups after controlling for age, sex, education level and smoking status. Intriguingly, of the four serum lipid indices, only HDL displayed a positive correlation with cognitive function, and this correlation was significantly weakened in the HD group that exhibited higher HDL level, as well as lower CHO, TG and LDL levels (see Fig. 1 and Table 2). This result supports the negative correlation between TG level and cognitive impairment in major depressive disorder^15^. However, the negative relationship between high lipoprotein levels and cognitive performance did not appear in the correlation matrix of the HD group, which may be due to our small sample size (n = 59), or a genuine group-wise discrepancy in serum lipid profile.

Notwithstanding a well-recognized association between lipoprotein and depressive symptoms, the specific roles of CHO, TG, HDL and LDL are not clearly known. Our results are mostly consistent with the previously proposed “unfavourable serum lipid profile” featuring higher HDL, lower LDL and CHO^24^. Previous findings suggest that depressive symptoms are associated with lower LDL level in adult women^18^ and men^24^, which are supported by our network analysis. However, contrary to studies that reported significant correlations of higher TG and CHO levels with depressive symptoms^15,32^, our network found that only HDL showed a positive association with self-rated depressive state.

A critical but unresolved issue therefore arises regarding whether HDL is protective against or contributes to depressive symptoms. We note that our finding of higher HDL level in the HD group contradicts with the results from most studies^27,32^. This inconsistency may reflect the complexity of HDL’s relationship with depression, although variations in methodology (different self-report scales, covariates or confounders considered) along with demographics and ethnicity of the sample studied, may also account for the discrepancy. Another reason for the inconsistent results might lie in the nature of the depressive spectrum. In this study, group differences in participants with higher SDS scores (above 50 points) and lower scores (below 50 points) were compared, while some other studies investigated the change of HDL between patients with clinically diagnosed major depressive disorder and healthy controls. For example, results from a larger sample in the Netherlands Study of Depression and Anxiety (NESDA) indicated lower HDL level in 761 participants currently with major depressive disorder than 629 healthy controls (*p* = 0.007)^32^. However, such significance disappeared after controlling for lifestyle-related factors such as BMI^32^. Therefore, it is important to recognize that adverse lifestyle behaviors (e.g. lower dietary fibre intake and smoking) may underlie serum lipid levels which consequently affect cognitive and mood symptoms^33^. Consistent with our hypothesis, major demographic and lifestyle confounding factors (i.e. age, sex, education level and smoking status) exhibited a significant effect on serum lipid levels, cognition, depressive status and their associations.

This study has several limitations. Firstly, despite the present findings suggesting a correlational pattern between self-reported depression and lipoproteins, this study did not empirically test the directionality of effect between depressive symptoms and serum lipid levels^24^. Secondly, due to difficulties in data collection, the comparison between depressive and non-depressive individuals in our samples was not matched for age and intelligence quotient (IQ), which might have biased our results. Further research would benefit from a larger and more demographically-balanced sample. Also, exclusion of participants with diagnosis of major depressive disorder is a major limitation for us to address the association between depressive symptoms, cognition and lipoprotein profile. Thus, future study should recruit participants with established major depressive disorder to examine whether a similar network pattern would be shown. Finally, our sample size was relatively small (n = 438) for network analysis, which limited our network’s stability. Future studies should investigate the relationship between increased HDL level and cognitive impairment in people with major depressive disorder and examine how this contributes to the core symptoms of major depressive disorder.

In summary, our results suggest that there may be distinct correlations between serum lipoprotein levels, cognitive performance and self-rated depressive status. The use of network analysis has added to the current knowledge in this field by illustrating the relationships between specific lipoprotein levels, cognitive function and self-rated depression after controlling for all other factors in the conceptual network. Serum lipoprotein levels may play a putative role in cognitive impairment in people with self-rated depression.

## Methods

### Participants

Participants were recruited from the annual physical examination event of the Wenzhou Kangning Hospital. A total of 575 people attended the event, and 512 people agreed to take part in the study upon invitation. Seventy-four participants were excluded due to physical health problems, taking regular medications and returning incomplete checklists. The final sample consisted of 438 adults, with 312 females and 126 males. The following inclusion criteria were applied: (1) staff of the hospital; (2) 18–70 years of age; (3) no history of mental illness that was diagnosed in the course of the physical and psychological examinations of newly recruited staff; (4) no history of alcohol and drug dependence; and (5) good past physical health. The study protocol was approved by the Ethics Committee of the Wenzhou Kangning Hospital, and all procedures were carried out in accordance with the approved guidelines and regulations. Written informed consent was obtained from each participant.

### Measurements

Cognitive performance was assessed using the RBANS^34^. The RBANS consists of 12 subtests that can be generalized into five sub-scale scores and a total score. The five sub-scales are (1) Immediate Memory (consisting of list learning and story memory tests); (2) Visuospatial/Constructional (consisting of figure copying and line orientation tests); (3) Language (consisting of picture naming and semantic fluency tests); (4) Attention (consisting of digit span and coding tests); and (5) Delayed Memory (consisting of list recall, story recall, figure recall and list recognition tests). Participants had to complete all tests to obtain a total cognitive score. The present study adopted the Chinese version of the RBANS that has been shown to have good test–retest reliability and validity in a healthy Chinese population^35^.

The SDS is a 20-item checklist which assesses the depressive state of participants^36^. The original SDS had a sensitivity of 97%, a specificity of 63%, and an 82% correct classification rate in distinguishing between depressed and non-depressed individuals. The responses were rated from 0 to 3 and summed to obtain a total score, which was then transformed into a standard score^37^. Based on a morbidity cutoff index from previous research^38^, participants who had a SDS standard score below 50 points were classified into the LD group, and those with a score above 50 were classified into the HD group in the present study.

### Serum lipoprotein levels

The overnight fasting blood sample was collected from all participants by venipuncture, between 7:00 and 9:00 am. The sample of approximately 5 ml venous blood was allowed to clot at room temperature, and then centrifuged at 3500 rpm for four minutes. After collecting the serum, the sample was stored at − 80 °C until it was thawed for assay. CHO, HDL, LDL and TG levels were measured by enzymatic colourimetry with a HITACHI 7180 automatic biochemistry analyzer (Hitachi High-Technologies Corporation, Japan) and commercially available kits (Medical System Biotechnology, Ningbo, China).

### Network estimation and statistical analysis

Network analysis is a new tool that visualizes partially controlled relational links among conceptualized factors^39^. In this study, we used the R software (version 3.5.0, available at https://cran.r-project.org/) to construct a partial correlation network encompassing data from all participants (n = 438). A total of 11 nodes representing: (1) lipid profile encompassing CHO, HDL, LDL, and TG levels; (2) a self-rated depressive state indexed by the SDS total score; and (3) cognitive performance level indexed by the total score and scores on the five sub-scales of the RBANS, were included to explore the relationships among lipoprotein levels, self-rated depressive state and cognitive performance of the participants. Two measures were applied to estimate network inference: Strength Centrality and Expected Influence (EI). Strength Centrality measures the number of direct connections of one node in the whole network, which connotes the importance of a particular node. One step EI is the sum of all edges of a node, while two-step EI measures the accuracy and stability of the constructed network. The bootstrapped difference test, the edge weight accuracy test, and the edge weight difference test were applied to all node centrality indices. The stability of the centrality estimates was also quantified by the correlation-stability-coefficient^40^.

To analyze the differences in lipoprotein levels and cognitive performance between the LD and HD groups, multivariate ANOVA was conducted while controlling for four major confounding factors (i.e. age, sex, education level and smoking status) using SPSS 16.0 (https://www.ibm.com/analytics/spss-statistics-software). Due to the small number of participants in the HD group (n = 59), we applied an additional partial correlation analysis to investigate the between-group differences in the influence of serum lipid levels on cognitive performance.

## Supplementary information

Supplementary Information.
