# Supplementary material for: The role of lipoprotein profile in depression and cognitive performance: a network analysis
Source: Sci Rep. 2020 Nov 26;10:20704. doi: 10.1038/s41598-020-77782-9 (PMC7693273; doi:10.1038/s41598-020-77782-9)
Supplement: Supplementary file 1 — Supplementary Information. [file 41598_2020_77782_MOESM1_ESM.docx]

**Title Page:**

**The role of lipoprotein profile in depression and cognitive performance: A network analysis**

Qiu-fang Jia ^1†^, Han-xue Yang ^2†^, Nan-nan Zhuang ^1†^, Xu-yuan Yin ^1^, Zhen-hua Zhu ^1^, Ying Yuan ^1^, Xiao-li Yin ^3^, Yi Wang ^2^, Eric F. C. Cheung ^4^, Raymond C. K. Chan ^2, 5,^ *, Li Hui ^1,^ *

^1^ The Affiliated Guangji Hospital of Soochow University, Medical College of Soochow University, Suzhou, Jiangsu, PR China

^2^ Neuropsychology and Applied Cognitive Neuroscience Laboratory, CAS Key Laboratory of Mental Health Institute of Psychology, Beijing, PR China

^3^ Wenzhou Kangning Hospital, Wenzhou Medical University, Wenzhou, Zhejiang, PR China

^4^ Castle Peak Hospital, Hong Kong, PR China

^5^ Department of Psychology, University of Chinese Academy of Sciences, Beijing, PR China

**Running Title:** The role of lipoprotein in depression and cognition

^†^ These three authors (Q.F.J, H.X.Y and N.N.Z.) contributed equally to this study.

*****Correspondence and requests for materials should be addressed to R.C.K.C (email：[rckchan@psych..ac.cn)](mailto:rckchan@psych..ac.cn)) and L.H. (email: huili004100@126.com).

**Supplementary Materials**

(A)


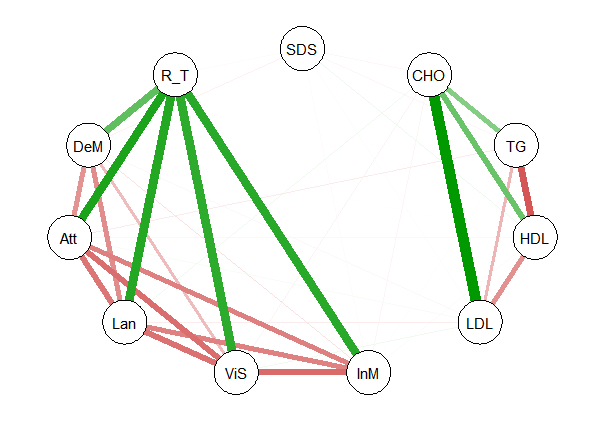


(B)


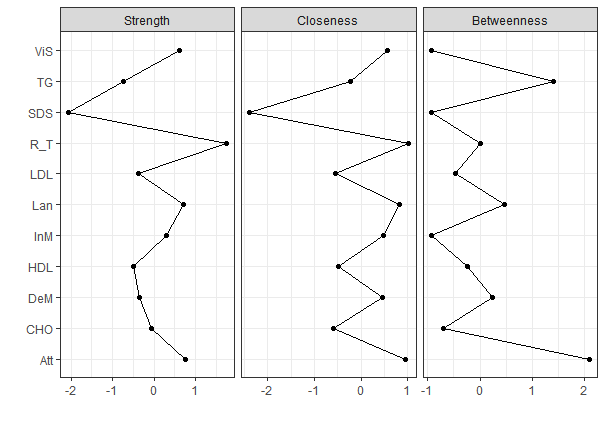


(C)


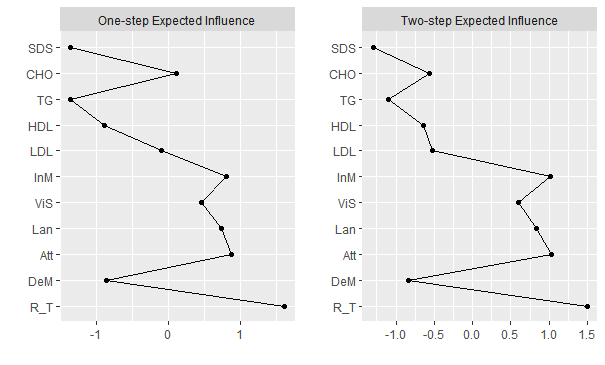


**Supplementary Figure1. (A) Regularized partial correlation network for the whole study samples (n=438); (B) Centrality plot of strength, closeness and betweenness of the partial correlation network; (C) Expected influence indexes of the network.**

**Note:** Green connections represent positive associations, whereas red connections represent negative associations. Thicker edge (positive and negative) indicates stronger partial correlations. **Att:** Attention sub-scale of the RBANS; **InM:** Immediate Memory sub-scale of the RBANS; **ViS**: Visual spatial /Constructional sub-scale of the RBANS; **Lan**: Language sub-scale of the RBANS; **DeM**: Delayed Memory sub-scale of the RBANS; **R_T**: RBANS total score; **CHO**: total cholesterol; **HDL**: high-density lipoprotein cholesterol; **LDL**: low-density lipoprotein cholesterol; **TG**: triglycerides; **SDS**: total score of the Self-Rating Depressive Scale.

***Supplementary Figure 1 (A)*** shows the estimated network (also LASSO-ed) without community identification. Centrality estimation of all nodes is displayed in ***Supplementary Figure 1 (B)***. Since strength index measures the number of direct connections of one node in the whole network, it connotes the importance of a certain node. SDS had the highest standardized strength centrality in the network, followed by R_T, both of which also had the highest closeness estimate in the network, suggesting that they had the shortest path connecting them to other nodes. This result was confirmed by EI indexes in ***Supplementary Figure 1 (C).***

(A)


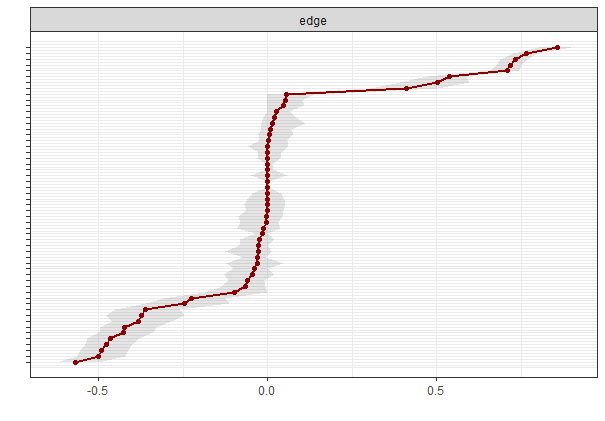


(B)


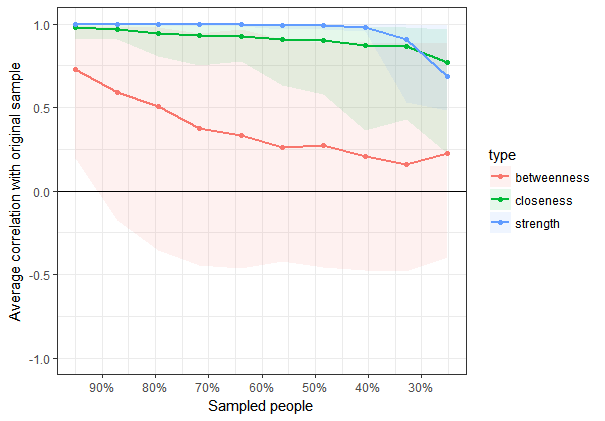


(C)


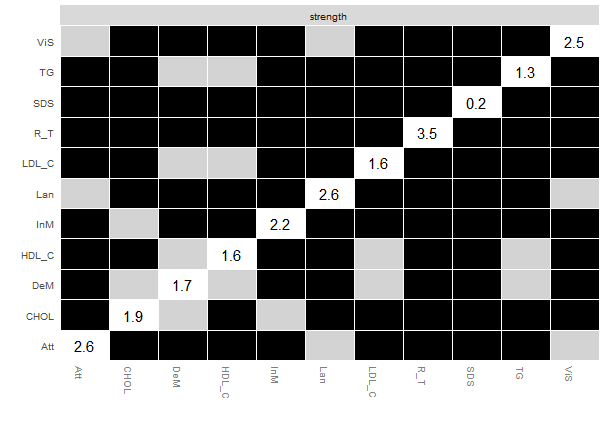


**Supplementary Figure2. (A) Bootstrapped confidence intervals of estimated edge-weights for the estimated network of 11 nodes.** The red line indicates the sample values and the gray area the bootstrapped CIs. Each horizontal line represents one edge of the network, ordered from the edge with the highest edge-weight to the edge with the lowest edge weight. In the case of ties (multiple edges-weights were estimated to be exact 0), the mean of the bootstrap samples was used in ordering the edges. The y-axis labels have been removed to avoid cluttering; **(B) Average correlations between centrality indices of networks sampled with cases dropped.** Lines indicate the means and areas indicate the range from the 2.5 quantile to the 97.5 quantile, there are sharp droppings of betweenness, but closeness and strength remained stable; **(C) Bootstrapped difference test (α=0.05) node strength of the 11 nodes.** Grey boxes indicate nodes that do not differ significantly from one-another and black boxes represent nodes that do differ significantly from one-another. White boxes show the value of node strength.

**Notes: Att:** Attention sub-scale of the RBANS; **InM:** Immediate Memory sub-scale of the RBANS; **ViS**: Visual spatial /Constructional sub-scale of the RBANS; **Lan**: Language sub-scale of the RBANS; **DeM**: Delayed Memory sub-scale of the RBANS; **R_T**: RBANS total score; **CHO**: total cholesterol; **HDL**: high-density lipoprotein cholesterol; **LDL**: low-density lipoprotein cholesterol; **TG**: triglycerides; **SDS**: total score of the Self-rating Depressive Scale.
